# Prognostic significance of overexpression of c-Met oncoprotein in cholangiocarcinoma

**DOI:** 10.1038/bjc.2011.199

**Published:** 2011-06-14

**Authors:** M Miyamoto, H Ojima, M Iwasaki, H Shimizu, A Kokubu, N Hiraoka, T Kosuge, D Yoshikawa, T Kono, H Furukawa, T Shibata

**Affiliations:** 1 Division of Cancer Genomics, National Cancer Center Research Institute, 5-1-1, Tsukiji, Chuo-ku, Tokyo 104-0045, Japan; 2 Division of Gastroenterologic and General Surgery, Department of Surgery, Asahikawa Medical College, Asahikawa, Japan; 3 Division of Molecular Pathology, National Cancer Center Research Institute, Tokyo, Japan; 4 Epidemiology and Prevention Division, Research Center for Cancer Prevention and Screening, National Cancer Center, Tokyo, Japan; 5 Hepatobiliary and Pancreatic Surgery Division, National Cancer Center Hospital, Tokyo, Japan

**Keywords:** c-Met, cholangiocarcinoma, immunohistochemistry, prognostic factor, epidermal growth factor receptor

## Abstract

**Background:**

Cholangiocarcinoma (CC) is a highly malignant carcinoma. We attempted to clarify the prognostic significance of c-Met overexpression and its association with clinicopathological factors in patients with CC.

**Patients and methods:**

One hundred and eleven patients with intrahepatic CC (IHCC) and 136 patients with extrahepatic CC (EHCC) who had undergone curative surgery were divided immunohistologically into c-Met^high^ and c-Met^low^ groups. Clinicopathological factors and outcomes were compared between the groups. c-Met and epidermal growth factor receptor (EGFR) expression was also examined in 10 CC cell lines.

**Results:**

The positivity of c-Met was 45.0% in IHCC and 68.4% in EHCC; c-Met^high^ expression was demonstrated in 11.7% of IHCC and 16.2% of EHCC. c-Met^high^ expression was significantly correlated with the 5-year survival rate for CC overall (*P*=0.0046) and for IHCC (*P*=0.0013), histopathological classification in EHCC, and for EGFR overexpression in both IHCC and EHCC. Coexpression and coactivation of c-Met and EGFR were also observed in CC cell lines. Multivariate analysis revealed that c-Met^high^ expression was an independent predictor of poor overall and disease-free survival in patients with IHCC.

**Conclusions:**

c-Met overexpression is associated with EGFR expression and is a poor prognostic factor in CC.

Cholangiocarcinoma (CC) is a highly malignant invasive carcinoma arising through malignant transformation of cholangiocytes. Epidemiologic studies have demonstrated that the incidence and mortality rates of this disease, especially those of intrahepatic CC (IHCC), are increasing worldwide (Mouzas *et al*, 2002; Okuda *et al*, 2002; Blechacz and Gores, 2008; Hezel and Zhu, 2008; Yachimski and Pratt, 2008; Aljiffry *et al*, 2009).

It is difficult to diagnose CC at an early stage because of the lack of clinical symptoms at this point, and most patients have unresectable disease at clinical presentation. Surgical resection is the only curative therapy, but among those patients who receive it, recurrence rates are high (Hezel and Zhu, 2008). To date, no randomised study has demonstrated any clear survival benefit of a specific chemotherapeutic regimen for cases of unresectable and recurrent CC (Aljiffry *et al*, 2009). Existing phase II data and a more recent meta-analysis suggest that gemcitabine and gemcitabine-based platinum regimens offer a slight advantage over other regimens (Hezel and Zhu, 2008).

Recently, a new treatment strategy for CC has been proposed, in the light of better understanding of the molecular mechanisms of carcinogenesis: it has been proposed that receptor tyrosine kinases (RTKs), such as epidermal growth factor receptor (EGFR), vascular epithelial growth factor (VEGF) and c-Met, are promising targets for treatment of CC (Socoteanu *et al*, 2008; Yoshikawa *et al*, 2008). In a previous report, we have indicated that EGFR and VEGF could be promising molecules for targeted therapy of CC (Yoshikawa *et al*, 2008, 2009).

c-Met, also known as scatter factor, is a high-affinity receptor for hepatocyte growth factor (HGF). Activation of HGF-c-Met signalling initiates cell invasiveness and triggers metastasis through direct involvement of tumour angiogenesis (Zhang *et al*, 2003). Upon ligand binding, c-Met activates multiple downstream signal transduction pathways, including the Grb2-Ras-mitogen-activated protein kinase (MAPK) cascade, the phosphatidylinositol-3 kinase (PI3K) pathway, and the signal transducer and activator of transcription (STAT) pathway (Weidner *et al*, 1993; Furge *et al*, 2000). c-Met partners include the integrin *α*6*β*4, CD44, plexin B, Fas and other RTKs such as RON, EGFR and ErbB2 (Gentile *et al*, 2008).

c-Met and EGFR are considered to assemble oncogenic signalling networks. Amplified c-Met activates members of the EGFR family and, conversely, mutated or amplified EGFR activates c-Met *in vitro* (Guo *et al*, 2008). EGFR is frequently coexpressed with c-Met in cell lines of lung, head and neck, breast, colon, and brain tumours (Reznik *et al*, 2008).

Enhanced expression of c-Met protein has been described in various solid tumours such as breast cancer (Garcia *et al*, 2007; Eder *et al*, 2009), oesophageal adenocarcinoma (Herrera *et al*, 2005), gastric cancer (Drebber *et al*, 2008; Ji *et al*, 2008), colon cancer (Liu *et al*, 1992), lung cancer (Lutterbach *et al*, 2007; Nakamura *et al*, 2007), ovarian cancer (Sawada *et al*, 2007), brain tumour (Kong *et al*, 2009), hepatocellular carcinoma (Boix *et al*, 1994; Suzuki *et al*, 1994), and biliary tract carcinoma (Terada *et al*, 1998; Hida *et al*, 1999; Aishima *et al*, 2002; Nakazawa *et al*, 2005). Recently, it has been proposed that c-Met might be a promising target for treatment of CC (Socoteanu *et al*, 2008). However, no study has yet demonstrated its prognostic significance in CC.

To improve our understanding of the clinical significance of c-Met in CC, the primary aim of this study is to clarify the frequency of c-Met overexpression. Following with this analysis, the second aim of this study is to analyse its association with clinicopathological factors, along with molecular data (EGFR, HER2, and VEGF expression), in the largest cohort (111 cases of IHCC and 136 cases of extrahepatic CC (EHCC)) of surgical specimens of CC. We also examined the expression of c-Met and EGFR in CC cell lines.

## Patients and methods

### Patients

A total of 247 patients with CC were examined in the present study. The patients had undergone surgery and been diagnosed histologically as having adenocarcinoma of the bile duct, except for cancer of gallbladder and ampulla of Vater, at the National Cancer Center Hospital, Tokyo, between February 1990 and July 2005. Patients who had other malignancies or had died within four weeks after surgery were excluded. Clinical and pathological data were obtained from the medical records of the patients. To examine the correlations of c-Met with other RTKs (EGFR, HER2, or VEGF), qualified cases including previous data for overexpression of these molecules (Yoshikawa *et al*, 2008) were examined.

The studied patients included 168 men and 79 women ranging in age from 33 to 82 years (median 65 years), who had been observed for periods ranging from 1.4 to 204.5 months (median 29.8 months). The cases were divided into two groups, IHCC and EHCC, in accordance with the TNM Classification of Malignant Tumours (Sobin and Wittekind, 2002) defined by the Union for International Cancer Control (UICC) and the World Health Organization Histological Classification of Tumours (Hamilton and Altonen, 2000). There were 111 cases of IHCC and 136 cases of EHCC. In this study, peri-hilar EHCC and distal EHCC are combined as EHCC because it is difficult to categorise EHCC based on the origin of the cystic duct. Tumour recurrence was defined as tumour growth in any site of the body after the operation, which was diagnosed clinically, radiologically, or pathologically, but mainly by computed tomography and ultrasonography. Only tumour death was used for analysis. The research protocol was approved by the Ethics Committee of the National Cancer Center, Tokyo, Japan. All patients gave written informed consent for inclusion in this study.

### Immunohistochemistry

Immunohistochemistry (IHC) was performed on 247 formalin-fixed, paraffin-embedded tissue sections. Immunohistochemical staining for c-Met was performed using a polymer-based method (Envision+Dual link-system-HRP (Dako, Glostrup, Denmark)). Serial sections (4 *μ*m thick) cut from representative paraffin-embedded serial tissue slices were prepared on silicone-coated slides for IHC evaluation. Sections cut through the maximum tumour diameter were selected for IHC evaluation. The sections were deparaffinised in xylene, and rehydrated through graded concentrations of ethanol (50–100%). Endogenous peroxidase activity was blocked by incubation in 0.3% hydrogen peroxide solution for 30 min. The antigens were retrieved by heating in a pressure cooker at 121°C for 10 min in 0.01 M citrate buffer. The tissue sections were incubated overnight at 4°C with anti-c-Met primary antibody (rabbit polyclonal; IBL, Gunma, Japan) at a dilution of 1 : 50. After a washing in PBS, the sections were treated with Envison+ Dual link reagent at room temperature for 30 min. 3,3′-Diaminobenzidine tetrahydrochloride was used as the chromogen, and the tissue sections were counterstained with haematoxylin.

Intensities of c-Met immunoreactivity were defined as: 0, complete absence of membrane staining or membrane staining in less than 30% of cancer cells; 1+, faint and partial membrane staining in at least 30% of cancer cells; 2+, strong and complete staining in at least 30% of cancer cells. The cases were divided into two groups, c-Met^low^ (0 or 1+) or c-Met^high^ (2+), for purposes of statistical analysis. The sections were evaluated by three observers, MM, HO, and TS, without knowledge of the clinical data. HO and TS are board-certified pathologists. IHC of EGFR and assessment of its expression were done as described previously (Yoshikawa *et al*, 2008).

### Cell lines

NCC-CC1, NCC-CC3-1, NCC-CC3-2, and NCC-CC4 cells were established from human IHCC, and NCC-BD1and NCC-BD2 from human EHCC, at the National Cancer Center Research Institute (Ojima *et al*, 2010). TKKK, HuCCT1, OZ, TGBC24TKB, and MKN45 were purchased from RIKEN Bio Resource Center or from the Japanese Collection of Research Bioresources. TKKK, TGBC24TKB, and HuCCT1 were established from IHCC, and OZ was from EHCC. MKN45 was a gastric cancer cell line that was used as a positive control, because of its high expression of c-Met and phospho-Met (Smolen *et al*, 2006). All of the cell lines had been derived from Japanese patients. The originally established six CC cell lines, HuCCT1 and MKN45 were maintained in RPMI with 10% bovine serum. TGBC24TKB, TKKK, and OZ were maintained in Dulbecco's modified Eagle medium with 10% bovine serum.

### Western blotting

Subconfluent cells were lysed at 4°C for 30 min using lysis buffer containing 10 mM Tris-HCl (pH 7.5), 1% Triton X-100, and 150 mM NaCl with a complete protease inhibitor cocktail (Roche, Basel, Switzerland) and a phosphate inhibitor cocktail (Nacalai Tesque, Kyoto, Japan). The protein concentration was determined using a Bio-Rad protein assay kit (Bio-Rad Laboratories, Hercules, CA, USA). Lysates (5 *μ*g protein per well) were separated by SDS-PAGE, then transferred to polyvinylidene difluoride membranes (Millipore, Billerica, MA, USA). The membranes were blocked with 5% skim milk in PBS for 30 min and then probed with the following primary antibodies: anti-c-Met (rabbit polyclonal; IBL; 1 : 1000), anti-phospho-Met (pY1234/1235, rabbit monoclonal, clone D26; Cell Signaling Technology, Danvers, MA, USA; 1 : 1000), anti-EGFR (mouse monoclonal, clone 31G7; Zymed, South San Francisco, CA, USA; 1 : 1000), and anti-phospho EGFR (pY1173, rabbit monoclonal, clone 53A5; Cell Signaling Technology) at 4°C overnight. After washing with PBS-Tween 20 (0.5%), the membranes were re-blocked and then incubated at room temperature for 1 h with horseradish peroxidase-conjugated goat anti-mouse or anti-rabbit antibody at a dilution of 1 : 1000. Following three washes, bands were visualised using the ECL Western Blotting Detection Reagents (GE Healthcare UK Ltd, Buckinghamshire, England). Anti-*β*-actin (mouse monoclonal; clone AC-15, Sigma, St Louis, MO, USA) was used as a loading control.

### Statistics

Correlations between the results of IHC and clinicopathological factors were determined by Fisher's exact probability test, except for histopathological classification, which was analysed by *χ*^2^-test. Cumulative survival rates and survival curves were calculated by the Kaplan–Meier method, and log-rank test was performed for the comparison of survival curves between low and high groups defied by c-Met expression level. The Cox proportional hazards model was used to estimate the hazard ratio and 95% confidence interval of each outcome (tumour death and recurrence). Multivariate analysis was performed for factors selected as risk factors by univariate analysis, except for UICC pT and UICC stage, which are composed of other factors. Correlations between the intensity of c-Met and that of EGFR in IHC or Western blotting were determined by Spearman's rank correlation. Statistical analysis was done using the Statview 5.0 statistical software package (Abacus Concepts, Berkeley, CA, USA). The level of significance was set at *P*<0.05.

## Results

### Immunohistochemical analysis of c-Met in human CC specimens

**Figure 1:**
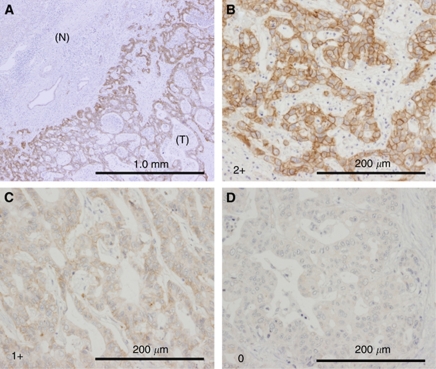
c-Met expression in primary CC cases. (**A**) c-MET expression was exclusively detected in tumour cells (T), but not in non-cancerous bile duct epithelium (N). (**B**–**D**) Representative IHC pictures of higher magnification of c-Met expression (expression score is 2+ (**B**), 1+ (**C**), and 0 (**D**), respectively). c-MET is localised in both the cell membrane and cytoplasm of CC cells. Scale bar indicates 1.0 mm (**A**) and 200 *μ*m (**B**–**D**).

**Figure 2:**
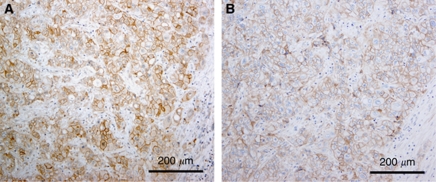
A representative case showing coexpression of c-Met (**A**) and EGFR (**B**) in adjacent sections of the same tumour. Scale bar indicates 200 *μ*m.

c-Met staining was localised in both the cell membrane and cytoplasm of CC cells (Figure 1). Strong immunostaining for c-Met was apparent at the luminal cell surface of neoplastic glands and ducts of adenocarcinoma. Positive staining for c-Met was demonstrated in 143 (57.9%, 95% CI: 51.7–64.1) of the 247 cases of CC overall, 50 (45.0%, 95% CI: 35.7–54.3) of the 111 cases of IHCC, and 93 (68.4%, 95% CI: 60.6–76.2) of the 136 cases of EHCC; high c-Met expression (2+) was demonstrated in 35 (14.2%, 95% CI: 9.8–18.6) of the 247 cases of CC overall, 13 (11.7%, 95% CI: 5.7–17.7) of the 111 cases of IHCC, and 22 (16.2%, 95% CI: 10.0–22.4) of the 136 cases of EHCC. When compared with EGFR staining, we occasionally observed coexpression of c-Met and EGFR (Figure 2).

### c-Met and EGFR expression in CC cell lines

**Figure 3:**
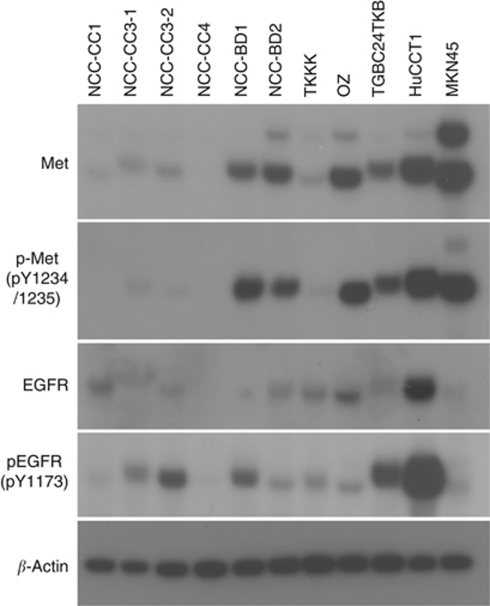
Immunoblot analysis of c-Met, phosphorylated-Met pY1234/1235), EGFR, and phosphorylated EGFR (pY1173) in CC cell lines. MKN45 cell (a human gastric cancer cell) is a positive control of c-Met and phosphorylated-Met expression (Smolen *et al*, 2006). *β*-actin is a loading control.

Expression of c-Met, phospho-Met, EGFR, and phospho-EGFR in ten CC cells and one gastric cancer cells were estimated by Western blotting (Figure 3). Expression of c-Met was observed in nine CC cells. Coexpression of c-Met and EGFR was detected in eight of them (except NCC-CC3-1). Prominent c-Met phosphorylation was detected in five cell lines (HuCCT1, OZ, NCC-BD2, TGBC24TKB, and NCC-BD1) and simultaneous activation of c-Met and EGFR was observed in seven cell lines including these five.

### Correlations between c-Met and clinicopathological factors

The relationships between c-Met expression and clinicopathological factors of IHCC and EHCC were evaluated and are shown in Tables 1 and 2. Increased expression of c-Met was significantly correlated with overexpression of EGFR in IHCC (*P*=0.0063), and histopathological classification (*P*=0.0239) and overexpression of EGFR (*P*=0.0056) in EHCC. No other clinical factors were associated with c-Met expression.

**Figure 4:**
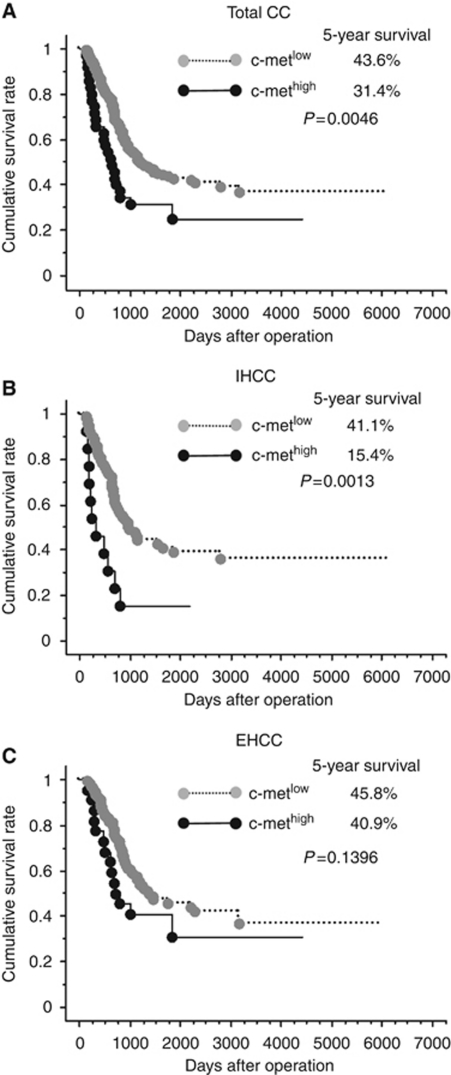
Survival curves according to c-Met expression. High c-Met expression was significantly correlated with poor survival in patients with CC as a whole (**A**) and in those with intrahepatic CC (IHCC) (**B**), but not in those with extrahepatic CC (EHCC) (**C**).

Five-year survival for patients in the c-Met^high^ and c-Met^low^ groups was 15.4 and 41.1% (*P*=0.0013) for IHCC and 40.9 and 45.8% (*P*=0.1396) for EHCC, respectively (Figure 4). We then performed multivariate analysis to assess the prognostic significance of c-Met expression. In IHCC, the independent predictors of poor overall survival were high c-Met expression (HR:3.92, 95% CI:1.62–9.48), macroscopic type (HR:4.57, 95% CI:1.44–14.51), intrahepatic metastasis (HR:3.27, 95% CI:1.78–5.99), and lymph node metastasis (HR:1.99, 95% CI:1.11–3.59). High c-Met expression (HR:3.50, 95% CI:1.56–7.85), macroscopic type (HR:4.78, 95% CI:1.69–13.4), intrahepatic metastasis (HR:2.78, 95% CI:1.60–4.82), lymph node metastasis (HR:2.94, 95% CI:1.70–5.08), venous invasion (HR:4.62, 95% CI:1.13–18.8), and EGFR overexpression (HR:1.98, 95% CI:1.12–3.51) were significant predictors of disease-free survival (Table 3).

In EHCC, the c-Met^high^ group tended to have a poor 5-year survival rate, but not to a significant degree. Univariate analysis also showed that c-Met^high^ was not a significant factor for survival. Therefore, multivariate analysis was not performed for EHCC.

## Discussion

In the present study, we have demonstrated the importance of c-Met overexpression in the prognosis and treatment of CC. We found that c-Met expression was correlated with EGFR overexpression in CC, and that it was also a significant prognostic factor in IHCC. In previous studies, the frequency of c-Met overexpression ranged from 21 to 58% in IHCC (Terada *et al*, 1998; Aishima *et al*, 2002; Nakazawa *et al*, 2005) and from 0 to 80% in EHCC (Hida *et al*, 1999; Nakazawa *et al*, 2005). This rather broad range is probably attributable to the small numbers of cases studied, or to differences in the definition of positivity. Moreover, no correlation between c-Met overexpression and clinical outcome of CC has been demonstrated previously. Here we showed that increased expression of c-Met was significantly associated with decreased overall and disease-free survival in patients with IHCC. The reason why c-Met expression was not a prognostic factor in EHCC may be partly explained by variables associated with their anatomic behaviour and methods of surgery.

Simultaneous expression of c-Met and EGFR has been observed in clinical specimens of primary chordoma (Weinberger *et al*, 2005) and gastrinoma (Peghini *et al*, 2002). Accumulated evidence has suggested that cross-talk occurs between c-Met and EGFR in several cancer cell lines (Jo *et al*, 2000; Farazi *et al*, 2006; Guo *et al*, 2008). Here we showed that c-Met expression was correlated with EGFR expression in clinical specimen of CC. We found that both EGFR and c-Met are broadly activated in CC cell lines. Eight CC cells coexpressed both c-Met and EGFR and coactivation of both proteins was detected in seven CC cell lines. It has been proposed that amplified c-Met drives the activity of EGFR family members and that mutated and amplified EGFR can drive c-Met activity (Guo *et al*, 2008). Mutual or unidirectional interaction between EGFR and MET activation has been reported in several cell lines (Bergstrom *et al*, 2000; Jo *et al*, 2000; Reznik *et al*, 2008). It is thought that either c-Met or EGFR stands at the top of the hierarchy of the downstream signalling pathway governed by the two molecules in a subset of cancer.

Collectively, it seems reasonable that efficient molecular therapy for CC should target multiple kinases such as c-Met, EGFR, and VEGFR. c-Met activation is regarded as one of the molecular mechanisms involved in the acquisition of resistance to anti-EGFR therapy, as activation of the alternative RTK pathway would bypass the EGFR pathway (Dempke and Heinemann, 2009). Therefore, inhibition of c-Met, either alone or in combination with an EGFR inhibitor, may be clinically beneficial in the setting of EGFR inhibitor resistance (Eder *et al*, 2009). Several studies have focused on combination therapy with c-Met inhibitors and agents targeting EGFR family members (Toschi and Janne, 2008).

In conclusion, c-Met overexpression is significantly correlated with overexpression of EGFR in CC and with prognosis in IHCC. Further molecular investigation of the interaction between EGFR and c-Met in this fatal disease is urgently needed.

## Figures and Tables

**Table 1:** Comparison of clinicopathological factors between patients with high and low c-Met expression in IHCC

	**c-Met**		
	**High**	**Low**	***P*-value**
*Gender*			
Male	7	59	0.7636
Female	6	39	
			
*Age*			
⩾65	9	48	0.2396
< 65	4	50	
			
*Tumour size*			
⩾5 cm	8	39	0.2430
<5 cm	5	52	
			
*Macroscopic type*			
Mass forming	10	83	0.4397
Non-mass forming	3	15	
			
*Intrahepatic metastasis*			
Negative	8	70	0.5229
Positive	5	28	
			
*Invasion to hepatic vein*			
Negative	5	53	0.2496
Positive	8	41	
			
*Invasion to portal vein*			
Negative	1	24	0.2907
Positive	12	73	
			
Lymph node metastasis			
Negative	7	57	0.7739
Positive	6	41	
			
*Histopathological classification*			
Well differentiated	4	21	0.5943
Moderately differentiated	8	73	
Poorly differentiated	1	4	
			
*UICC pT*			
*In situ*+1+2a+2b	4	28	>0.9999
3+4	9	70	
			
*UICC stage*			
I+II	8	51	0.5680
III+IVA	5	47	
			
*Lymphatic vessel invasion*			
Negative	2	37	>0.9999
Positive	11	61	
			
*Venous invasion*			
Negative	1	19	0.4566
Positive	12	79	
			
*Perineural invasion*			
Negative	4	27	0.7536
Positive	9	71	
			
*Hepatic surgical margin*			
Negative	9	84	0.2202
Positive	4	14	
			
*Bile duct margin*			
Negative	10	86	0.3797
Positive	3	12	
			
*EGFR expression*			
Negative	5	72	0.0063
Positive	8	21	
			
*VEGF expression*			
Negative	7	51	0.5697
Positive	6	42	
			
*HER2 expression*			
Negative	13	92	>0.9999
Positive	0	1	

Abbreviations: EGFR*=*epidermal growth factor receptor; IHCC=intrahepatic CC; UICC=Union for International Cancer Control; VEGF=vascular epithelial growth factor.

**Table 2:** Comparison of clinicopathological factors between patients with high and low c-Met expression in EHCC

	**c-Met**		
	**High**	**Low**	**>P-value**
*Gender*			
Male	16	86	0.7914
Female	6	28	
			
*Age*			
⩾65	16	59	0.1004
<65	6	55	
			
*Tumour size*			
⩾3 cm	11	63	0.8144
<3 cm	10	50	
			
*Macroscopic type*			
Polypoid	3	19	>0.9999
Non-polypoid	18	91	
			
*Depth of tumour invasion*			
Within fm	2	13	>0.9999
Beyond fm	20	101	
			
*Invasion to hepatic artery*			
Negative	21	111	0.5106
Positive	1	3	
			
*Invasion to portal vein*			
Negative	20	82	0.0649
Positive	2	32	
			
*Lymph node metastasis*			
Negative	10	65	0.3554
Positive	12	49	
			
*Histopathological classification*			
Papillary	4	18	0.0239
Well differentiated	2	30	
Moderately differentiated	9	55	
Poorly differentiated	7	11	
			
*Lymphatic vessel invasion*			
Negative	2	98	0.7369
Positive	20	16	
			
*Venous invasion*			
Negative	3	18	>0.9999
Positive	19	96	
			
*Perineural invasion*			
Negative	4	21	>0.9999
Positive	18	93	
			
*Dissected periductal structures margin*			
Negative	18	97	0.7480
Positive	4	17	
			
*Bile duct margin*			
Negative	16	82	>0.9999
Positive	6	32	
			
*Invasion to other organ*			
Negative	12	44	0.2363
Positive	10	70	
			
*EGFR expression*			
Negative	12	93	0.0056
Positive	9	16	
			
*VEGF expression*			
Negative	7	46	0.4798
Positive	14	63	
			
*HER2 expression*			
Negative	19	100	>0.9999
Positive	2	9	

Abbreviations: EGFR*=*epidermal growth factor receptor; EHCC=extrahepatic CC; fm=fibromuscular layer; VEGF=vascular epithelial growth factor.

**Table 3:** Multivariate analyses of overall survival and disease-free survival in patients with IHCC. (Cox proportional hazards model)

	**Overall survival**			**Disease-free survival**		
	**HR**	**95% CI**	***P*-value**	**HR**	**95% CI**	***P*-value**
*Macroscopic type*						
Mass forming	4.572	1.440–14.516	0.0099	4.783	1.698–13.470	0.0030
Non-mass forming	1.00			1.00		
						
*Intrahepatic metastasis*						
Negative	1.00			1.00		
Positive	3.270	1.783–5.999	0.0001	2.781	1.604–4.822	0.0003
						
*Invasion to portal vein*						
Negative	1.00			—		
Positive	0.881	0.388–1.999	0.7623	—	—	—
						
*Lymph node metastasis*						
Negative	1.00			1.00		
Positive	1.998	1.110–3.597	0.0209	2.947	1.707–5.088	0.0001
						
*Histopathological classification*						
Well differentiated	1.00			1.00		
Moderately differentiated	1.507	0.639–3.554	0.3491	0.753	0.345–1.642	0.4759
Poorly differentiated	2.031	0.526–7.835	0.3036	1.199	0.340–4.227	0.7772
						
*Lymphatic vessel invasion*						
Negative	1.00			1.00		
Positive	3.119	0.851–11.435	0.0860	2.723	0.759–9.768	0.1243
						
*Venous invasion*						
Negative	1.00			1.00		
Positive	3.121	0.825–11.807	0.0935	4.628	1.136–18.854	0.325
						
*Perineural invasion*						
Negative	1.00			1.00		
Positive	0.588	0.265–1.305	0.1917	0.511	0.244–1.072	0.756
						
*Bile duct margin*						
Negative	1.00			—		
Positive	1.871	0.902–3.882	0.0926	—	—	—
						
*EGFR expression*						
Negative	1.00			1.00		
Positive	1.745	0.957–3.180	0.0690	1.987	1.125–3.511	0.0180
						
*c-Met expression*						
Negative	1.00			1.00		
Positive	3.921	1.620–9.487	0.0003	3.502	1.562–7.851	0.0023

Abbreviations: CI=Confidence interval; EGFR*=*epidermal growth factor receptor; HR=Hazard ratio; IHCC=intrahepatic CC.
